# Dietary supplementation with potassium-magnesium sulfate modulates the antioxidant capacity, immunity, and gut microbiota in weaned piglets

**DOI:** 10.3389/fmicb.2022.961989

**Published:** 2022-08-23

**Authors:** Shuting Cao, Kaiyong Huang, Xiaolu Wen, Jingchun Gao, Bailei Cui, Kang Yao, Xianliang Zhan, Shenglan Hu, Qiwen Wu, Hao Xiao, Cui Zhu, Zongyong Jiang, Li Wang

**Affiliations:** ^1^State Key Laboratory of Livestock and Poultry Breeding, Ministry of Agriculture Key Laboratory of Animal Nutrition and Feed Science in South China, Guangdong Key Laboratory of Animal Breeding and Nutrition, Maoming Branch, Guangdong Laboratory for Lingnan Modern Agriculture, Institute of Animal Science, Guangdong Academy of Agricultural Sciences, Guangzhou, China; ^2^School of Life Sciences and Engineering, Foshan University, Foshan, China

**Keywords:** potassium-magnesium sulfate, growth performance, antioxidant capacity, gut microbiota, weaned piglets

## Abstract

The purpose of this study was to evaluate the effects of different levels of potassium magnesium sulfateon (PMS) on growth performance, diarrhea rate, intestinal morphology, antioxidant capacity, intestinal immunity, and gut microbiota in weaned piglets. A total of 216 weaned piglets were randomly divided into six dietary groups: the basal diet with 0% (CON), 0.15, 0.3, 0.45, 0.6, and 0.75% PMS. The results showed that the ADFI of 29–42 days and 1–42 days was linearly and quadratically increased by the PMS supplementation (*P* < 0.05), and significantly reduced the diarrhea rate in weaned piglets (*P* < 0.05). Moreover, dietary supplementation with PMS significantly reduced the serum adrenaline and noradrenaline levels in weaned piglets (*P* < 0.05). Furthermore, 0.3% PMS significantly increased the activity of glutathione peroxidase (GSH-Px) in the jejunum (*P* < 0.05) and tended to increase the activity of superoxide dismutase (SOD) in the jejunal mucosa of piglets (*P* < 0.1). Additionally, dietary supplementation with PMS significantly reduced the interleukin-1β (IL-1β) level in the jejunal mucosa (*P* < 0.05), and 0.3% PMS increased the serum IgM content in piglets (*P* < 0.05). Furthermore, the analysis of colonic microbiota by 16S RNA sequencing showed that the addition of PMS increased the Shannon index (*P* < 0.05) and Observed Species index (*P* < 0.05). Based on linear discriminant analysis effect size (LEfSe) and *T*-test analysis, the addition of PMS increased the relative abundance of *Ruminococcaceae* and *Peptostreptococcaceae* in the colonic digesta (*P* < 0.05). Spearman analysis showed that there was a positive correlation between intestinal GSH-Px activity and the relative abundance of *Peptostreptococcaceae*. These results showed that dietary supplementation with PMS could improve growth performance, alleviate diarrhea incidence, and modulate the antioxidant capacity and intestinal immunity in weaned piglets, which was partially related to the significant changes in colonic microbiota composition.

## Introduction

Weaning stress caused dysfunctions in metabolism, digestion, and immune responses in piglets when suddenly facing nutritional, immunological, and psychological challenges (Upadhaya and Kim, 2021). Weaning is accompanied by intestinal injury, disorders of digestion, antioxidant system dysfunction, immune imbalance, diarrhea, and even death of piglets (Moeser et al., 2017; Cao et al., 2018; Cao et al., 2022). Hence, finding an effective nutritional strategy to prevent antioxidant and immune systems disruption in weaning piglets is important for animal husbandry.

Potassium and magnesium, as the essential major elements for animals, are used as cations to regulate the body’s electrolyte balance in animals (Stone et al., 2016; Fiorentini et al., 2021). Potassium can regulate the appropriate osmotic pressure in cells and the acid-base balance of body fluids and participate in the metabolism of carbohydrates and proteins (Stone et al., 2016). The addition of potassium-containing compounds to drinking water could reduce the heat stress of animals (Ansari et al., 2020). Magnesium is involved in maintaining the stability of the nucleic acid structure, activating enzymes in the body, inhibiting nerve excitability, and participating in protein synthesis and muscle contraction (Fiorentini et al., 2021). Moreover, magnesium is an important activator of various enzyme systems in cell metabolism (Lötscher et al., 2022). Further, dietary magnesium supplementation improves lifespan and enhances antioxidant capacity in a mouse model of progeria (Villa-Bellosta, 2020). However, excessive addition of potassium and magnesium in animal diets led to renal function damage, electrolyte loss, and even hyperkalemia (Al Alawi et al., 2018; Gerhardt and Angermann, 2019). So, it remained unclear about the combination of potassium and magnesium on intestinal antioxidant capacity, immunity, and gut microbiota in weaned piglets.

Therefore, a natural compound mineral additive of potassium and magnesium (potassium-magnesium sulfate, PMS) was used to evaluate the effect of dietary supplementation with PMS on the growth performance, antioxidant capacity, intestinal immunity, and gut microbiota in weaned piglets.

## Materials and methods

### Animal ethics

All animal experimental protocols used in the current study were according to the Chinese guidelines for animal welfare and approved by the Animal Care and Use Committee of Guangdong Academy of Agricultural Sciences (GAASIAS-2016-017).

### Experimental animals, design, diets, and housing

A total of 216 piglets (Duroc × Landrace × Large White) weaned at 21 days with an initial average body weight of 7.53 kg, were randomly allotted into six dietary groups with six replicates of six piglets each replicate. Piglets were fed the basal diet supplemented with 0, 0.15, 0.3, 0.45, 0.6, and 0.75% PMS. The PMS products were obtained from Qinghai Lanhushancheng Bio-tech Co., Ltd. (Qinghai, China), which is composed of K_2_SO_4_.MgSO_4_.6H_2_O, Potassium: 21%, Magnesium: 6.5%. The potassium-magnesium sulfate ore was collected in the Qinghai Salt Lake, sorted by the screw machine, and removed the iron and plastic paper debris through a sieve. And then, the sorted potassium-magnesium sulfate ore was pulverized by a pulverizer and dried by the air-cooled dryer at a low temperature. Furthermore, the dried potassium-magnesium sulfate ore was further sorted by a 20-mesh sieve, and the particle size of potassium-magnesium sulfate ore smaller than 20 mesh was collected as the potassium-magnesium sulfate. The composition of the basal diets (Table 1) was formulated to meet or exceed the nutritional requirements for weaned piglets recommended by National Research Council (2012). All pigs were housed in an environmentally controlled room with slatted plastic flooring and an effective mechanical ventilation system. Each pen had two stainless feeders and four nipple drinkers. Pigs had *ad libitum* access to feed and water throughout the experimental period. The animal house temperature was controlled at 25–28°C and relative humidity was controlled at 55–65%. There were no antimicrobial and anticoccidial drugs used during the trial.

**TABLE 1:** Ingredient and energy composition of weaned piglets’ diets.

Item	1–28 days	29–42 days
**Ingredients, %**		
Corn	34.05	56.18
Expanded corn	12.00	13.00
Fermented soybean meal	10.00	10.00
Soybean meal	5.00	6.00
Expanded soybean	11.00	3.67
Fish meal	3.00	3.00
Whey powder	15.00	−
Whey protein concentrate	1.00	−
Soybean oil	1.50	1.00
White granulated sugar	2.00	2.00
Calcium citrate	1.40	−
Calcium carbonate	−	1.10
Calcium hydrogen phosphate	0.600	0.60
L-lysine hydrochloride	0.6	0.60
DL-Methionine	0.15	0.15
L-threonine	0.20	0.20
L-tryptophan	0.05	0.05
NaCl	0.30	0.30
50% Choline chloride	0.15	0.15
Premix^a^	2.00	2.00
Total	100	100
**Energy and nutrient composition,^b^ %**		
Digestive energy	3600	3490
Crude protein	18.7	17.2
SID Lys	1.44	1.35
SID Met	0.44	0.45
SID Thr	0.85	0.83
SID Trp	0.26	0.24
Calcium	0.70	0.76
Phosphorus	0.55	0.53
Available phosphorus	0.35	0.31
Magnesium	0.94	0.64
Potassium	0.15	0.13

^a^The premix provided the following in diets: VA 4, 400 IU*kg^–1^, VD_3_ 440 IU*kg^–1^, VE 30 IU*kg^–1^, VK 1 mg*kg^–1^, VB_12_ 40 μg*kg^–1^, VB_1_ 3 mg*kg^–1^, VB_2_ 10 mg*kg^–1^, Nicotinic acid 40 mg*kg^–1^, D-pantothenic acid 15 mg*kg^–1^, Folic acid 1 mg*kg^–1^, VB_6_ 8 mg*kg^–1^, Biotin 0.08 mg*kg^–1^, (FeSO_4_●H_2_O) 120 mg*kg^–1^, (CuSO_4_●5H_2_O) 16 mg*kg^–1^, (MnSO_4_●H_2_O) 70 mg*kg^–1^, (ZnSO_4_●H_2_O) 120 mg*kg^–1^, (CaI_2_●O_6_) 0.7 mg*kg^–1^, (Na_2_SeO_3_) 0.48 mg*kg^–1^.

^b^Nutrient levels were calculated values, except that the digestible energy, crude protein, and available phosphorus were determined values.

### Growth performance and diarrhea rate

Pigs were weighed individually on days 0, 24, and 42. The average daily feed intake (ADFI), average daily gain (ADG), and feed to gain ratio (F: G) were recorded and calculated. The Feed:Gain ratio (F:G) = (Feed intake)/(Weight gain). The diarrhea rate was recorded every day during this experiment and quantified according to previous methods (Tang et al., 2021).

### Sample treatment and collection

Piglets were weighed after fasting for 12 h at the end of the days 14, 28, and 42. After weighing at day 42, one piglet from each replicate was selected to slaughter. The blood was collected by the anterior vena cava. After collection of the whole blood, allow the blood to clot by leaving it undisturbed at room temperature for 30 min, and collect the serum by centrifuging at 3,000 ×*g* for 10 min in a refrigerated centrifuge. Subsequently, the duodenum, jejunum, ileum, cecum, and colon were quickly removed. About 2 cm segments of duodenum, jejunum, and ileum were immediately isolated and then fixed in 4% neutral paraformaldehyde solution. The mucosal samples from the jejunum were harvested by gently scraping with a glass slide and rapidly frozen in liquid nitrogen and stored at −80°C for further determinations. The colonic digesta samples were rapidly frozen in liquid nitrogen and stored at −80°C for the determination of gut microbiota composition and diversity.

### Intestinal histological analysis

The specimens of the intestinal segments were embedded in paraffin and cut into 4-mm thickness sections for H&E staining according to the methods previously described (Yang et al., 2014). The tissue sections were measured under a microscope using an image processing and analysis system (Leica, Germany). The Program Image-pro Plus 6 (Media Cybernetics, Inc., GA, United States) was used to determine the villus height (VH), crypt depth (CD), and villus height-to-crypt depth (VH:CD) ratio. At least 25 villus samples with intact lamina propria were blindly selected and measured.

### Analysis of antioxidant capacity in serum and intestine

Commercial kits were used to analyze the antioxidant capacity of serum and intestine mucosa, including activities of superoxide dismutase (SOD), total antioxidant capacity (T-AOC), catalase (CAT), glutathione peroxidase (GSH-Px), and malondialdehyde (MDA) content according to the manufacturer’s protocols (Nanjing Jiancheng Biotechnology, Nanjing, China). Reactive oxygen species (ROS) levels in serum and jejunum mucosa were determined according to the manufacturer’s instructions (R&D Systems, MN, United States).

### Analysis of immune factors in serum and intestine

The levels of immunoglobulin A (IgA), immunoglobulin M (IgM), immunoglobulin G (IgG), secretory immunoglobulin A (sIgA), interleukin-1β (IL-1β), tumor inflammatory factor-α (TNF-α), interleukin-8 (IL-8), and interleukin-10 (IL-10) were determined according to the manufacturer’s instructions (Enzyme-linked Biotechnology Co. Ltd., Shanghai, China), and measured with a multi-functional microplate reader (SynergyTM H1, BioTek, United States).

### 16S rRNA-based microbiota analysis

Total DNA was extracted from colonic digesta using a commercial kit according to the manufacturer’s instructions (Omega Bio-Tek, Norcross, GA, United States). The DNA concentration was checked by NanoDrop 2000 Spectrophotometer (Thermo Fischer Scientific, Wilmington, NC, United States), and the DNA quality was monitored by 1% agarose gel electrophoresis. The V3-V4 variable region of the 16S rRNA gene was amplified using the specific primers (341 F:5′-CCTAYGGGRBGCASCAG-3; 806 R:5′-GGACTACNNGGGTATCTAAT-3′). The PCR products were detected by agarose gel electrophoresis, and the 300 bp amplicon was cleaned and subjected to 16S rDNA sequencing on an Illumina HiSeq 2500 PE 250 platform (Novogene Bioinformatics Technology Co., Ltd., Tianjin, China). All sequence data processing was performed using the QIIME software package. Sequences were paired-end and high-quality sequences were aligned against the SILVA database (Ribocon GmbH, Bremen, Germany). The UCHIME software (Tiburon, CA, United States) was used to identify and remove chimeric sequences. Operational taxonomic units (OTUs) were assigned at a 97% identity using the SILVA database. The Venn diagram with shared and unique OTUs was used to identify the similarity and differences among treatments. The clustered OTUs were used to calculate the alpha-diversity within groups including Shannon index, Simpson index, Chao 1 richness, Good’s coverage, and Observed species of the whole tree. Beta diversity index, principal coordinate analysis (PCoA plots, weighted UniFrac distance), non-metric multidimensional scaling (NMDS plots, weighted UniFrac distance), and unweighted pair-group method with arithmetic means (UPGMA) clustering were accessed to calculate the β-diversity between groups. The differences in the relative abundances of microbiota among treatments were compared using the linear discriminant analysis effect size (LEfSe) and *T*-test analyses.

### Statistical analysis

Data were presented as mean ± SEM. All data were statistically analyzed by ANOVA using the LSD multiple comparisons by the SPSS 23 software after the Levene test and the Kolmogorov Smirnov test. *P* < 0.05 and 0.05 < *P* < 0.1 was considered as statistically significant and tendency, respectively.

## Results

### Growth performance and diarrhea rate

There are no differences in the initial body weight, final body weight, ADG, ADFI, and F:G of piglets between different PMS levels groups (*P* > 0.05) (Table 2). Piglets fed with 0.6% PMS tended to have higher ADFI at 29–42 days compared with other treatments (*P* < 0.1). Moreover, the ADFI of 29–42 days and 1–42 days was linearly and quadratically increased by the enhanced PMS level (*P* < 0.05). There were no animals that died during the experiment.

**TABLE 2:** Effect of dietary supplementation with PMS on growth performance in weaned piglets.

Item	PMS, %						*P*-value		
	0	0.15	0.30	0.45	0.60	0.75	ANOVA	Linear	Quadratic
**ADG, g/d**									
1–14 days	314 ± 19	314 ± 41	316 ± 32	316 ± 19	323 ± 38	318 ± 19	0.99	0.66	0.91
15–28 days	524 ± 30	532 ± 11	531 ± 13	521 ± 16	541 ± 34	540 ± 22	0.98	0.51	0.85
29–42 days	554 ± 20	600 ± 27	616 ± 15	594 ± 12	619 ± 31	607 ± 15	0.32	0.09	0.11
1–42 days	464 ± 24	482 ± 35	488 ± 10	477 ± 17	494 ± 42	488 ± 21	0.48	0.11	0.22
**ADFI, g/d**									
1–14 days	412 ± 10	411 ± 8	412 ± 13	412 ± 7	423 ± 10	420 ± 5	0.90	0.33	0.57
15–28 days	745 ± 34	762 ± 20	757 ± 35	750 ± 9	779 ± 25	777 ± 21	0.91	0.32	0.60
29–42 days	862 ± 26	960 ± 35	966 ± 13	941 ± 15	975 ± 47	963 ± 22	0.09	0.04	0.03
1–42 days	673 ± 20	711 ± 17	712 ± 10	701 ± 9	726 ± 22	720 ± 11	0.23	0.04	0.09
**F: G**									
1–14 days	1.31 ± 0.05	1.32 ± 0.05	1.31 ± 0.03	1.30 ± 0.02	1.32 ± 0.06	1.32 ± 0.02	0.99	0.87	0.93
15–28 days	1.43 ± 0.11	1.43 ± 0.06	1.42 ± 0.09	1.44 ± 0.08	1.46 ± 0.15	1.44 ± 0.06	0.99	0.61	0.88
29–42 days	1.56 ± 0.14	1.60 ± 0.09	1.57 ± 0.09	1.59 ± 0.07	1.58 ± 0.13	1.59 ± 0.05	0.98	0.83	0.96
1–42 days	1.45 ± 0.05	1.48 ± 0.04	1.46 ± 0.03	1.47 ± 0.04	1.47 ± 0.05	1.48 ± 0.02	0.80	0.33	0.60
**Diarrhea rate, %**									
1–14 days	4.36 ± 0.79	5.36 ± 1.05	2.78 ± 0.59	5.16 ± 1.18	4.96 ± 1.28	4.36 ± 0.79	0.49	0.89	0.98
15–28 days	8.13 ± 1.21	8.73 ± 1.29	5.16 ± 1.86	5.95 ± 1.92	4.56 ± 1.04	8.73 ± 0.25	0.13	0.48	0.11
29–42 days	8.53 ± 1.28^a^	7.93 ± 0.85^a^	4.36 ± 1.33^b^	4.76 ± 1.27^b^	4.56 ± 1.04^b^	3.97 ± 0.59^b^	0.02	0.00	0.00
1–42 days	7.01 ± 0.89	7.34 ± 0.85	4.10 ± 0.90	5.29 ± 0.95	4.69 ± 0.95	5.69 ± 0.20	0.05	0.07	0.04

ADG, Average daily gain; ADFI, Average daily feed intake; F: G, Feed to gain ratio.

^a,b^Within a row, means without a common superscript letter differ at P < 0.05. Data were presented as mean + SEM.

Additionally, supplementation with 0.3, 0.45, 0.6, and 0.75% PMS could significantly reduce the diarrhea rate in weaned piglets at 29–42 days (*P* < 0.05). The piglets in the 0.3% PMS group had a lower diarrhea rate in comparison with other groups (*P* < 0.05). From 1 to 42 days, the diarrhea rate was quadratically decreased by dietary PMS treatments (*P* < 0.05).

### Serum biochemical indices

As shown in Table 3, the PMS treatments tended to enhance the serum glucose content of weaned piglets (0.05 < *P* < 0.1). Moreover, the serum glucose content of weaned piglets showed a significant quadratic relationship with PMS levels (*P* < 0.05, Quadratic). However, the levels of total protein, creatinine, alanine aminotransferase, glutamic oxalacetic transaminase, alkaline phosphatase, albumin, blood urea nitrogen, urea, triglycerides, total cholesterol, high-density lipoprotein, and low-density lipoprotein were not significantly influenced by dietary PMS treatments (*P* > 0.05).

**TABLE 3:** Effect of dietary supplementation with PMS on serum biochemical indices in weaned piglets.

Item	PMS, %						*P*-value		
	0	0.15	0.30	0.45	0.60	0.75	ANOVA	Linear	Quadratic
TP, g/L	57.53 ± 2.89	60.9 ± 3.48	57.33 ± 1.42	60.4 ± 2.09	58.46 ± 1.33	57.38 ± 1.46	0.76	0.79	0.72
HDL, mmol/L	0.96 ± 0.06	0.99 ± 0.05	0.95 ± 0.06	0.94 ± 0.07	0.90 ± 0.06	0.91 ± 0.07	0.92	0.32	0.60
LDL, mmol/L	1.13 ± 0.19	1.05 ± 0.19	0.94 ± 0.13	1.11 ± 0.09	0.89 ± 0.09	1.02 ± 0.10	0.80	0.43	0.63
AST, U/L	73.68 ± 9.99	79.57 ± 16.35	53.88 ± 5.50	81.3 ± 15.37	80.2 ± 15.87	75.62 ± 10.59	0.67	0.72	0.87
ALP, U/L	215.08 ± 21.61	203.45 ± 35.42	166.45 ± 16.53	217.88 ± 46.95	214.53 ± 25.06	206.51 ± 11.84	0.81	0.86	0.83
ALB, g/L	29.59 ± 1.51	29.91 ± 2.21	29.74 ± 0.72	29.88 ± 1.41	27.74 ± 1.08	29.79 ± 1.78	0.91	0.66	0.91
BUN, mmol/L	1.93 ± 0.31	1.69 ± 0.09	1.73 ± 0.14	1.88 ± 0.29	1.80 ± 0.23	2.03 ± 0.21	0.89	0.58	0.54
UA, μmol/L	23.94 ± 3.16	37.5 ± 3.72	28.96 ± 4.90	32.04 ± 4.49	32.53 ± 6.80	32.34 ± 4.85	0.51	0.46	0.58
GLU, mmol/L	2.93 ± 0.22	2.00 ± 0.47	2.21 ± 0.39	2.38 ± 0.27	3.21 ± 0.42	3.19 ± 0.21	0.07	0.10	0.03
TG, mmol/L	0.62 ± 0.18	0.59 ± 0.05	0.45 ± 0.04	0.53 ± 0.09	0.50 ± 0.05	0.49 ± 0.03	0.73	0.21	0.37
TC, mmol/L	2.33 ± 0.15	2.33 ± 0.22	2.05 ± 0.15	2.24 ± 0.12	1.96 ± 0.11	2.12 ± 0.07	0.39	0.11	0.24
CRE, μmol/L	121.65 ± 12.07	113.40 ± 4.48	103.32 ± 3.86	103.19 ± 5.76	114.19 ± 4.78	113.27 ± 5.33	0.36	0.49	0.11
ALT, U/L	56.88 ± 4.07	47.42 ± 4.39	42.93 ± 1.97	52.48 ± 3.32	50.87 ± 4.21	51.20 ± 3.28	0.17	0.79	0.22

TP, Total protein; HDL, High-density lipoprotein; LDL, Low-density lipoprotein; AST, Osteocalcin; ALP, Alkaline phosphatase; ALB, Albumin; BUN, Blood urea nitrogen; UA, Urea; GLU, Glucose; TG, Triglycerides; TC, Total cholesterol; CRE, Creatinine; ALT, Alanine aminotransferase.

### Serum hormone contents

The dietary supplementation with PMS significantly reduced the contents of serum norepinephrine and epinephrine (Table 4) (*P* < 0.05). However, dietary PMS treatments did not affect the serum levels of cortisol and glucocorticoid (*P* > 0.05).

**TABLE 4:** Effect of dietary supplementation with PMS on serum hormone in weaned piglets.

Item	PMS, %						*P*-value		
	0	0.15	0.30	0.45	0.60	0.75	ANOVA	Linear	Quadratic
COR, ng/ml	1.50 ± 0.01	1.50 ± 0.01	1.51 ± 0.00	1.51 ± 0.00	1.50 ± 0.00	1.50 ± 0.01	0.68	0.84	0.45
NE, ng/ml	7.69 ± 0.53^a^	6.34 ± 0.37^b^	5.74 ± 0.39^b^	5.60 ± 0.46^b^	5.94 ± 0.23^b^	5.93 ± 0.32^b^	0.02	0.01	0.00
EP, ng/ml	5.11 ± 0.35^a^	4.39 ± 0.15^ab^	4.10 ± 0.18^b^	3.87 ± 0.29^b^	4.17 ± 0.22^b^	4.17 ± 0.30^b^	0.05	0.03	0.00
GC, ng/ml	3.89 ± 0.56	4.96 ± 0.58	3.75 ± 0.47	2.59 ± 1.02	3.66 ± 1.37	4.50 ± 1.04	0.57	0.79	0.62

COR, Cortisol; NE, Norepinephrine; EP, epinephrine; GC, Glucocorticoid.

^a,b^Within a row, means without a common superscript letter differ at P < 0.05. Data were presented as mean ± SEM.

### Intestinal morphology

As shown in Table 5, the ratio of villus height to crypt depth in the ileum showed a significant linear relationship with PMS levels (*P* = 0.05). Moreover, the depth of duodenal crypt depth showed a significant quadratic relationship with PMS levels in weaned piglets (*P* < 0.05). However, dietary PMS treatments did not affect the intestinal morphology of the duodenum, jejunum, and ileum (*P* > 0.05).

**TABLE 5:** Effects of dietary supplementation with PMS on intestinal morphology in weaned piglets.

Item	PMS, %						*P*-value		
	0	0.15	0.30	0.45	0.60	0.75	ANOVA	Linear	Quadratic
**Duodenum**									
Villus height	537 ± 43	532 ± 27	500 ± 19	490 ± 19	494 ± 20	514 ± 42	0.68	0.18	0.20
Crypt depth	365 ± 30	368 ± 25	341 ± 10	334 ± 26	315 ± 26	411 ± 20	0.11	0.76	0.03
V:C ratio	1.47 ± 0.23	1.40 ± 0.12	1.49 ± 0.09	1.51 ± 0.16	1.51 ± 0.08	1.44 ± 0.06	0.77	0.45	0.58
**Jejunum**									
Villus height	450 ± 21	479 ± 38	473 ± 50	449 ± 26	435 ± 10	450 ± 33	0.93	0.57	0.72
Crypt depth	269 ± 24	274 ± 15	242 ± 11	265 ± 15	277 ± 25	291 ± 25	0.64	0.39	0.23
V:C ratio	1.73 ± 0.14	1.77 ± 0.14	1.98 ± 0.22	1.70 ± 0.06	1.63 ± 0.14	1.56 ± 0.08	0.40	0.20	0.20
**Ileum**									
Villus height	387 ± 8	344 ± 13	375 ± 22	355 ± 17	370 ± 9	409 ± 27	0.15	0.26	0.04
Crypt depth	311 ± 24	325 ± 23	267 ± 13	269 ± 17	248 ± 16	305 ± 31	0.11	0.16	0.08
V:C ratio	1.27 ± 0.08	1.17 ± 0.07	1.41 ± 0.09	1.33 ± 0.09	1.52 ± 0.09	1.37 ± 0.09	0.14	0.05	0.57

V:C ratio: The ratio of villus height to crypt depth. Data were presented as mean ± SEM.

### Serum and intestinal antioxidant index

**FIGURE 1:**
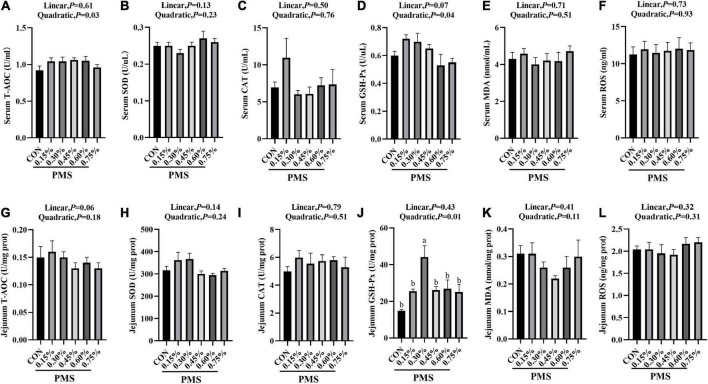
Effects of dietary supplementation with PMS on serum and intestinal antioxidant indexes in weaned piglets. **(A)** Serum T-AOC level; **(B)** Serum SOD activity; **(C)** Serum CAT activity; **(D)** Serum GSH-Px activity; **(E)** Serum MDA content; **(F)** Serum ROS content; **(G)** Jejunal T-AOC level; **(H)** Jejunal SOD activity; **(I)** Jejunal CAT activity; **(J)** Jejunal GSH-Px activity; **(K)** Jejunal MDA content; **(L)** Jejunal ROS content. T-AOC, Total antioxidant capacity; SOD, Total superoxide dismutase; CAT, Catalase; GSH-Px, Glutathione peroxidase; MDA, Malondialdehyde; ROS, Reactive oxygen species. CON: basal diet; 0.15%: the diet contained 0.15% PMS, 0.3%: the diet contained 0.3% PMS, 0.45%: the diet contained 0.45% PMS, 0.6%: the diet contained 0.6% PMS, 0.75%: the diet contained 0.75% PMS. PMS, Potassium-magnesium sulfate. ^a,b^Within a row, means without a common superscript letter differ at *P* < 0.05. Data were presented as mean ± SEM.

In Figure 1, 0.3% PMS significantly increased the activity of GSH-Px (Figure 1J) in the jejunum (*P* < 0.05) and tended to enhance the activity of GSH-Px (Figure 1D) in serum and SOD activity (Figure 1H) in the jejunum (*P* < 0.1). Moreover, the levels of T-AOC (Figure 1A), GSH-Px (Figure 1D) in serum and GSH-Px (Figure 1J) activity in jejunum showed a significant quadratic relationship with PMS levels (*P* < 0.05, Quadratic). And we found that there was no significant difference in the ROS contents in serum and jejunum of weaned piglets treated with different PMS levels (*P* > 0.05) (Figures 1F,L).

### Intestinal immune cytokines and immunoglobulin levels

**FIGURE 2:**
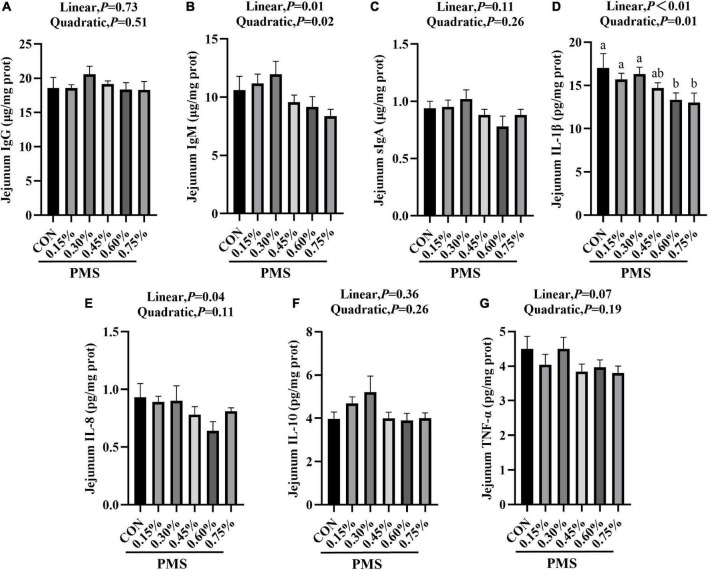
Effects of dietary supplementation with PMS on intestinal immune factors in weaned piglets. **(A)** IgG content; **(B)** IgM content; **(C)** sIgA content; **(D)** IL-1β content; **(E)** IL-8 content; **(F)** IL-10 content; **(G)** TNF-α content. IgG, Immunoglobulin G; IgM, Immunoglobulin M; sIgA, Secretory Immunoglobulin A; IL-1β, Interleukin-1β; IL-8, Interleukin-8; IL-10, Interleukin-10; TNF-α, Tumor inflammatory factor-α. CON: basal diet; 0.15%: the diet contained 0.15% PMS, 0.3%: the diet contained 0.3% PMS, 0.45%: the diet contained 0.45% PMS, 0.6%: the diet contained 0.6% PMS, 0.75%: the diet contained 0.75% PMS. PMS, Potassium-magnesium sulfate. ^a,b^Within a row, means without a common superscript letter differ at *P* < 0.05. Data were presented as mean ± SEM.

In Figure 2, the addition of 0.6 and 0.75% PMS significantly reduced IL-1β (Figure 2D) in the jejunum of weaned piglets (*P* < 0.05). The content of IgM (Figure 2B) reached the peak at 0.3% (*P* < 0.05, Linear, Quadratic), and decreased linearly with the increase of the addition of PMS.

### Microbial composition analysis by 16S RNA sequencing

**FIGURE 3:**
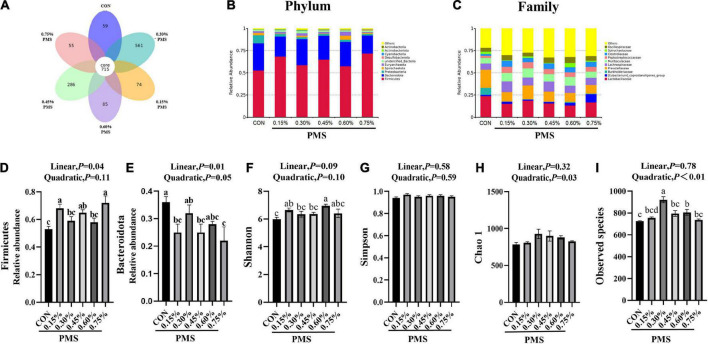
Effect of PMS supplementation on the composition and diversity index of colon microbiota in weaned piglets. **(A)** The common and special OTUs distribution among the different levels of PMS treatments is shown by the Petal diagram. **(B,C)** The relative abundance of top ten phyla **(B)** and families **(C)**. **(D,E)** The relative abundance of Firmicutes **(D)** and Bacteroidota **(E)**. **(F–I)** Alpha diversity index, including Shannon index **(F)**, Simpson index **(G)**, Chao1 index **(H)**, and observed species **(I)**. CON: basal diet; 0.15%: the diet contained 0.15% PMS, 0.3%: the diet contained 0.3% PMS, 0.45%: the diet contained 0.45% PMS, 0.6%: the diet contained 0.6% PMS, 0.75%: the diet contained 0.75% PMS. PMS, Potassium-magnesium sulfate. ^a,b,c^Within a row, means without a common superscript letter differ at *P* < 0.05. Data were presented as mean ± SEM.

There are 715 OTU clusters shared by the six groups, while 0.3% PMS has 561 unique OTU clusters (Figure 3A). The top 10 abundance of bacteria at the phylum (Figure 3B) included *Firmicutes*, *Bacteroidota*, *Proteobacteria*, *Spirochaetota*, *Euryarchaeota*, *Unified bacteria*, *Desulfobacterota*, *Cyanobacteriota*, *Actinobacterita*, and *Actinobacteria*. At the family level, the dominant top 10 bacteria are *Lactobacillaceae*, *Prevotellaceae*, *Eubacterium coprostanogenes group*, *Burkholderiaceae*, *Lachnospiraceae*, *Muribaculaceae*, *Peptostreptococcaceae*, *Clostridiaceae*, *Spirochaetaceae*, and *Oscillospiraceae* (Figure 3C). The addition of 0.15, 0.45, and 0.75% PMS significantly enhanced the abundance of *Firmicutes* and decreased the abundance of *Bacteroidota* (Figures 3D,E).

### Microbial α-diversity of the colon contents

Compared with the control group, the addition of 0.15 and 0.6% PMS significantly increased the Shannon index of colonic digesta (Figure 3F) in weaned piglets (*P* < 0.05). Moreover, the Simpson index (Figure 3G) was not influenced by the addition of PMS (*P* > 0.05), while the Chao1 index (Figure 3H) was quadratically increased with the addition of PMS (*P* < 0.05). The addition of 0.30% and 0.60% PMS significantly increased the observed species index (*P* < 0.05) (Figure 3I).

### Microbial composition analysis by 16S RNA sequencing

**FIGURE 4:**
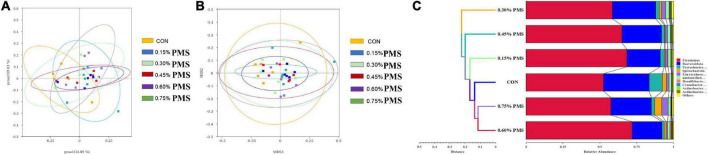
Effect of PMS supplementation on the β-diversity of colon microbiota in weaned piglets. **(A,B)** PCoA plot **(A)** and NMDS plot **(B)** based on ASV weighted unifrac distance. **(C)** UPGMA clustering was conducted based on unweighted unifrac distance. CON: basal diet; 0.15%: the diet contained 0.15% PMS, 0.3%: the diet contained 0.3% PMS, 0.45%: the diet contained 0.45% PMS, 0.6%: the diet contained 0.6% PMS, 0.75%: the diet contained 0.75% PMS. PMS, Potassium-magnesium sulfate.

The effects of PMS on the β-diversity of colonic microbiota in weaned piglets are shown in Figure 3. The PCoA (Figure 4A) and NMDS plots (Figure 4B) based on weighted UniFrac distance were applied to evaluate the microbial β-diversity. As revealed by both PCoA and NMDS plots, the microbial community structure of different samples is not clearly separated among different groups (Figures 4A,B). The UPGMA clustering based on unweighted UniFrac showed significant differences in the microflora composition of colonic contents among the six groups (Figure 4C). In particular, *Firmicutes* in the PMS addition group had significantly prominent clusters compared with the control group (Figure 4C).

### LEfse analysis and *T*-test analysis

**FIGURE 5:**
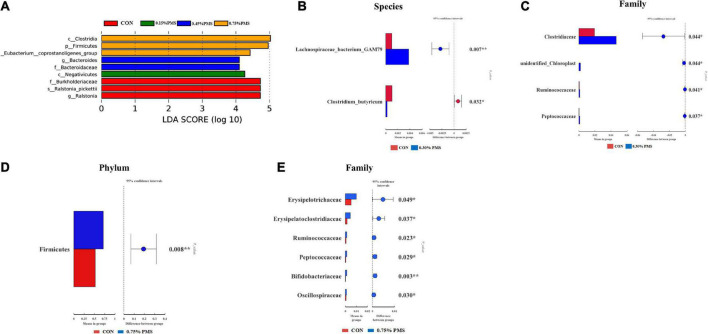
LEfSe analysis and *T*-test analysis for the significant changes of differential colon microbiota at different levels in weaned piglets. **(A)** The LEfSe analysis (LDA score ≥ 4) identified the biomarker bacterial species in the six groups. **(B,C)** Group A and C different microbiota *T*-test analyses based on Species **(B)** and Family **(C)** levels. **(D,E)** Group A and F different microbiota *T*-test analysis based on Phylum **(D)** and Family **(E)** levels. CON: basal diet; 0.15%: the diet contained 0.15% PMS, 0.3%: the diet contained 0.3% PMS, 0.45%: the diet contained 0.45% PMS, 0.6%: the diet contained 0.6% PMS, 0.75%: the diet contained 0.75% PMS. PMS, Potassium-magnesium sulfate. *P < 0.05 and **P < 0.01.

The differential microbiota from different treatments is presented based on LEfSe analysis. The results in Figure 5A show that the control group was enriched with *Ralstonia* (genus), *Ralstonia pickettii* (species), *Burkholderiaceae* (family). On the other hand, 0.15% PMS addition group is enriched with *Negativicutes* (class), 0.45% PMS addition group is enriched with *Bacteroidaceae* (family), *Bacteroides* (genus), 0.75% PMS addition group is enriched with *[Eubacterium] coprostanogenes group*, *Firmicutes* (phylum), and *Clostridia* (class). In Figure 5B, *T*-test analysis results showed that the 0.3% PMS addition group increased the relative abundance of *Lachnospiraceae bacterium GAM79* at the boundary level (*P* < 0.05) and decreased the relative abundance of *Clostridium*, *Butyricum* compared with the control group (*P* < 0.05). Moreover, the relative abundance of *Clostridiaceae*, *Unidentified Chloroplast*, *Ruminococcaceae*, and *Peptococcaceae* was increased by 0.3% PMS addition at the family level (*P* < 0.05) (Figure 5C). Compared with the control group, the 0.75% PMS addition group increased the relative abundance of Firmicutes at the phylum level (*P* < 0.05), as well as *Erysipelotrichaceae*, *Erysipelatoclostridiaceae*, *Ruminococcaceae*, *Peptococcaceae*, *Bifidobacteriaceae*, and *Oscillospiraceae* at the family level (*P* < 0.05) (Figures 5D,E).

### Spearman correlation analysis

**FIGURE 6:**
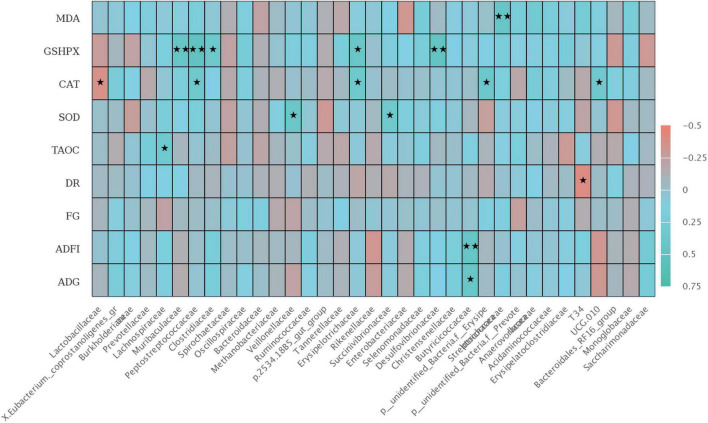
The Spearman correlation analysis of colon microbial species at the family level with ADG, ADFI, F: G, MDA, GSH-Px, CAT, SOD, and T-AOC differential metabolites in weaned piglets. The heatmap with red indicated a negative correlation, while blue represented a positive correlation. ADG, Average daily gain; ADFI, Average daily feed intake; F: G, Feed to gain ratio; TAOC, total antioxidant capacity of jejunum; SOD, total superoxide dismutase of jejunum; CAT, catalase of jejunum; GSH-Px, glutathione peroxidase of jejunum; MDA, malondialdehyde of jejunum. ^★^*P* < 0.05 and ^★★^*P* < 0.01.

There was a significant correlation between colonic microbiota at the family level with growth performance and jejunal antioxidant capacity of weaned piglets (Figure 6). The relative abundance of *Butyriccoccaceae* was positively correlated with ADFI (*P* < 0.01) and ADG during 1–42 days (*P* < 0.05), and the relative abundance of *T34* was negatively correlated with diarrhea rate (*P* < 0.05). Furthermore, T-AOC activity in jejunal mucosa was positively correlated with *Lachnospiraceae* (*P* < 0.05). SOD activity in jejunal mucosa was positively correlated with the relative abundances of *Veillonellaceae*, *Succenivibrionaceae*, and *Streptcoccaceae* (*P* < 0.05). The GSH-Px activity was positively correlated with the relative abundances of *Muribaculaceae*, *Peptostreptococcaceae*, *Desulfovibrionaceae* (*P* < 0.01), and *Clostridiaceae*, *Erysipelotrichaceae* (*P* < 0.05). Interestingly, there was a negative correlation in CAT activity with the relative abundances of *Lactobacillaceae* and *Erysipelotrichaceae* (*P* < 0.05), but positively correlated with *UCG 010* (*P* < 0.05).

## Discussion

Weaning often results in intestinal digestion and absorption dysfunction, diarrhea, and even death, which caused huge economic losses in livestock husbandry (Upadhaya and Kim, 2021). We found that the addition of different doses of PMS linearly increased the ADFI and ADG, and significantly reduced the diarrhea rate of weaned piglets. Magnesium and potassium levels were found to affect the appetite, growth, and behaviors of the animals (Adam and Dawborn, 1972; McCaughey and Tordoff, 2002; Laires et al., 2004). A previous study reported that oral administration of magnesium by capsule to lambs resulted in the restoration of appetite, but intravenous injection of magnesium sulfate had a lesser effect (Martin et al., 1964). Livingston et al. (2019) Broilers fed supplemental potassium diets trended toward an improved feed conversion ratio from 29 to 35 days compared to broilers fed basal potassium deficiency diets (Livingston et al., 2019). So, we speculate that the addition of PMS in the feed enhanced the appetite of weaning piglets, and further led to the enhanced average daily feed intake of piglets. In this study, the addition of different doses of PMS linearly increased the ADFI and ADG, and significantly reduced the diarrhea rate of weaned piglets. Similarly, previous studies reported that supplemental magnesium significantly increased the piglet’s weight at birth (Zang et al., 2014). Moreover, Plush et al. (2018) found that maternal magnesium sulfate supplementation in a pre-farrow diet improves factors important for piglet viability (Plush et al., 2018). Under stressful conditions, the hypothalamus pituitary adrenal (HPA) axis is activated (Sarkodie et al., 2019), leading to an enhancement in cortisol and catecholamine stress hormone secretion (Pulopulos et al., 2020). In this study, we found that dietary addition of PMS significantly reduced the contents of serum norepinephrine and epinephrine, suggesting that PMS may alleviate the weaning stress of piglets.

Weaning stress leads to the excessive production of oxygen free radicals, resulting in lipid peroxidation and damage to the structure and function of biofilm (Cao et al., 2018; Degroote et al., 2020; Novais et al., 2021). A previous study reported that the exogenous addition of magnesium can alleviate the decrease of GSH-Px activity in mitochondria of premature aging mice caused by oxidative stress (Villa-Bellosta, 2020). Morais et al. (2017) showed that magnesium plays a key role in several metabolic reactions, particularly in oxidative stress in obese individuals (Morais et al., 2017). Similarly, we found that the addition of 0.3% PMS significantly increased the GSH-Px activity and tended to increase the SOD activity of the jejunum of weaned piglets, indicating that PMS could improve the intestinal antioxidant capacity and alleviate the intestinal oxidative stress in piglets during weaning.

Cytokines such as the interleukin family play a critical role in immune stress. Especially, circulating IL-1β, IL-6, and IL-8 are upregulated in systemic and chronic inflammatory conditions (Bester and Pretorius, 2016). Xie et al. (2019) reported that magnesium isoglycyrrhizinate suppresses LPS-induced inflammation and oxidative stress by inhibiting NF-κB and MAPK pathways in RAW264.7 cells (Xie et al., 2019). Lopez-Baltanas et al. (2021) found that magnesium supplementation reduces inflammation in rats with induced chronic kidney disease (Lopez-Baltanas et al., 2021). Moreover, magnesium sulfate differentially modulates fetal membrane inflammation in a time-dependent manner (Cross et al., 2018). In this experiment, we found that dietary addition of PMS linearly reduced the inflammatory factor IL-1β and IL-8 levels in the jejunum. Immunoglobulin is one of the components of the immune system of animals (Cao et al., 2022). Newborn piglets lack protective immunity and they mainly rely on the absorption of immunoglobulin from colostrum to obtain passive immunity (Levast et al., 2014; Cao et al., 2022). Some studies have found that the anionic salt in the sow diet is positively related to the content of immunoglobulin (Wang et al., 2020; Yang et al., 2022). So far, the information regarding the effects of PMS on immunoglobulin levels and cytokines levels in weaning pigs is limited. We found that the addition of PMS had a trend to enhance IgM in the intestine, which may be due to the increase of dietary cations for stimulating the expression of immunoglobulin by the addition of PMS in weaned piglet diet.

Intestinal barrier dysfunction and immune-mediated injury are usually related to the disruption of the host gut microbial composition. We found that the addition of PMS significantly increased the relative abundance of Firmicutes, and decreased the abundance of Bacteroidetes. Previous research found that the increased feed intake of weaning pigs may be related to the change in gut microbiota structure (Ban-Tokuda et al., 2017), with Firmicutes being the dominant bacteria affecting pig feed efficiency (Myer et al., 2015). Moreover, the data showed that the addition of PMS increased the Shannon index, Observed Species, Simpson index, and Chao1 index in the colonic microbiota of weaned piglets. Similarly, Crowley et al. (2018) found that the addition of magnesium-rich marine minerals significantly improved the intestinal microbial diversity of adult male rats (Crowley et al., 2018). Further Lefse and *T*-test analysis showed that PMS improved antioxidant and intestinal immunity. However, high-dose magnesium supplements can lead to the decline of microbial diversity and ecological imbalance in rats (García-Legorreta et al., 2020). Our results showed that the relative abundance of *Clostridiaceae*, *Unidentified chloroplast*, *Ruminococcaceae*, and *Peptostreptococcaceae* in the 0.3% PMS group was higher than that in the control group. Furthermore, the 0.75% PMS group increased the relative abundance of *Erysipelotrichaceae*, *Erysipelatoclostridiaceae*, *Ruminococcaceae*, *Peptostreptococcaceae*, *Bifidobacteria CEAE*, and *Oscillospiraceae* in the family level compared with the control group. A previous study reported that the enhanced *Peptostreptococcaceae* abundance in rat intestines is related to the reduced oxidative stress induced by a high-fat diet (Yang et al., 2019). Consistently, we found that PMS increased the abundance of *Peptostreptococcaceae*, which may involve alleviating the weaning stress of piglets.

The gut immune and antioxidant homeostasis are associated with distinct alternations in the intestinal microbiome (Ghaisas et al., 2016; Gershon and Margolis, 2021). A previous study found that *Erysipelotrichaceae* may worsen intestinal inflammation by enhancing the response of Th17 cells and molecular simulation of myelin oligodendrocyte glycoprotein (MOG) (Maghzi and Weiner, 2020). In this study, the addition of high-dose PMS leads to the increase of *Erysipelotrichaceae*, which may affect intestinal health and growth performance. Spearman’s analysis showed that there was a correlation between intestinal GSH-Px was positively correlated with *Muribaculaceae*, *Peptostreptococcaceae*, and *Desulfovibrionaceae*, suggesting that PMS may affect the activity of GSH-Px by increasing the abundance of *Peptostreptococcaceae*. Several studies reported that intestinal flora and antioxidant capacity are interrelated (Tomasello et al., 2016; Wang et al., 2017). According to the previous studies, Yi et al. (2021) found that the MDA (liver) was positively correlated with *Erysipelotrichaceae* in colon microorganisms and negatively correlated with *Ruminococcus* through the protective effect of antrodina on alcohol-induced intestinal microbiota and liver metabolomic disorders in mice (Yi et al., 2021). The results show that intestinal microorganisms can affect the antioxidant ability of the different organs. Miao et al. (2021) found that it enhanced the GSH-Px activity in the small intestine of mice and increased the relative abundance of *Lactobacillus* after the 5 ml/kg walnut oil gavage test on mice (Miao et al., 2021). In our experiment, we analyzed the correlation between intestinal oxidative stress and colonic microbial changes and found that there was a correlation between intestinal GSH-Px was positively correlated with *Muribaculaceae*, *Peptostreptococcaceae*, and *Desulfovibrionaceae*, suggesting that PMS may affect the activity of GSH-Px by increasing the abundance of *Peptostreptococcaceae*. This finding can provide a potential target for improving the antioxidant capacity of weaned piglets.

### Conclusion

In conclusion, dietary supplementation with PMS could improve the growth performance, antioxidant activity, and immune capacity in weaned piglets, which was partially related to the significant changes in colonic microbiota composition. Thus, PMS may be a potential feed additive to improve growth performance and intestinal health of weaning piglets.

## Data availability statement

The datasets presented in this study can be found in online repositories. The names of the repository/repositories and accession number(s) can be found in the article/supplementary material.

## Ethics statement

This animal study was reviewed and approved by Animal Care and Use Committee of Guangdong Academy of Agricultural Sciences.

## Author contributions

SC, KH, and XW: investigation, methodology, data curation, validation, visualization, and writing—original draft. SH, BC, and JG: visualization, validation, and formal analysis. KY, XZ, QW, and HX: investigation, methodology, formal analysis, and resources. LW, ZJ, CZ, and SC: conceptualization, supervision, funding acquisition, and writing—review and editing. All authors contributed to the article and approved the submitted version.
